# Automated Lung-Related Pneumonia and COVID-19 Detection Based on Novel Feature Extraction Framework and Vision Transformer Approaches Using Chest X-ray Images

**DOI:** 10.3390/bioengineering9110709

**Published:** 2022-11-18

**Authors:** Chiagoziem C. Ukwuoma, Zhiguang Qin, Md Belal Bin Heyat, Faijan Akhtar, Abla Smahi, Jehoiada K. Jackson, Syed Furqan Qadri, Abdullah Y. Muaad, Happy N. Monday, Grace U. Nneji

**Affiliations:** 1 School of Information and Software Engineering, University of Electronic Science and Technology of China, Chengdu 610054, China; 2 IoT Research Center, College of Computer Science and Software Engineering, Shenzhen University, Shenzhen 518060, China; 3 Centre for VLSI and Embedded System Technologies, International Institute of Information Technology, Hyderabad 500032, India; 4 Department of Science and Engineering, Novel Global Community Educational Foundation, Hebersham, NSW 2770, Australia; 5 School of Computer Science and Engineering, University of Electronic Science and Technology of China, Chengdu 610054, China; 6 School of Electronic and Computer Engineering, Peking University Shenzhen Graduate School, Peking University, Shenzhen 518060, China; 7 Research Center for Healthcare Data Science, Zhejiang Lab, Hangzhou 311121, China; 8 IT Department, Sana’a Community College, Sana’a 5695, Yemen

**Keywords:** lung disease, COVID-19, pneumonia, chest X-rays images, feature extraction, automatic detection, artificial intelligence, epidemic

## Abstract

According to research, classifiers and detectors are less accurate when images are blurry, have low contrast, or have other flaws which raise questions about the machine learning model’s ability to recognize items effectively. The chest X-ray image has proven to be the preferred image modality for medical imaging as it contains more information about a patient. Its interpretation is quite difficult, nevertheless. The goal of this research is to construct a reliable deep-learning model capable of producing high classification accuracy on chest x-ray images for lung diseases. To enable a thorough study of the chest X-ray image, the suggested framework first derived richer features using an ensemble technique, then a global second-order pooling is applied to further derive higher global features of the images. Furthermore, the images are then separated into patches and position embedding before analyzing the patches individually via a vision transformer approach. The proposed model yielded 96.01% sensitivity, 96.20% precision, and 98.00% accuracy for the COVID-19 Radiography Dataset while achieving 97.84% accuracy, 96.76% sensitivity and 96.80% precision, for the Covid-ChestX-ray-15k dataset. The experimental findings reveal that the presented models outperform traditional deep learning models and other state-of-the-art approaches provided in the literature.

## 1. Introduction

Lung disease is widespread across the globe. Chronic disease, tuberculosis, asthma, pneumonia, fibrosis, and other diseases fall into this category. A new coronavirus disease (COVID-19) has now been causing major respiratory problems and breathing issues since early December 2019. It has been claimed that about 63.2 million individuals have been infected globally, with around 1.47 million fatalities. The World Health Organization (WHO) is constantly providing nations with the knowledge they need to protect themselves against COVID-19 [1]. Infected people with COVID-19 experience medium to mild symptoms such as shortness of breath, cough, and fever. However, several persons died as a result of severe pneumonic diseases in their lungs [2,3]. The majority of those who died from COVID-19 had severe chest constriction (pneumonia) as a result of a considerable decrease in oxygen levels, which also led to a catastrophic heart attack [4]. Pneumonia, on the other hand, is a type of lung disease that causes congestion in the tiny air sacs inside the human body’s lungs. The lungs may get clogged with a large amount of fluid, making breathing difficult. Pneumonia can be caused by viruses (flu or COVID-19), bacterial infections or the common cold. Because of the emergence of COVID-19 sickness, medical specialists are finding it difficult to diagnose lung infections from chest X-ray images [5].

X-ray, magnetic resonance imaging (MRI), isotope, computed tomography (CT), and other medical imaging [6,7,8,9] methods have long been used to diagnose lung disorders. Radiologists and clinicians routinely utilize X-ray and CT images to diagnose lung disorders. As a result, many doctors, particularly during the COVID-19 time, advocate a chest X-ray for lung illness analysis [10]. Medical practitioners have employed X-ray imaging for many decades to assess and investigate numerous anomalies in human body organs [11]. Many studies have shown that the X-ray technique is a cost-effective tool for illness detection, giving pathological changes as well as economic productivity and non-invasive qualities. Lung infections have been observed in chest X-ray images as blunted costophrenic angles, consolidations, cavitation, infiltrates and widely dispersed nodules [12]. As a result, radiologists use X-ray images to diagnose illnesses such as pneumonia, infiltration, nodule, fractures, pleurisy, pneumothorax, pericarditis and effusion [13].

Detecting and classifying lung disorders using chest X-ray images is a difficult task for radiologists. As a result, researchers focused heavily on developing automated lung disease detection tools. Many computer-aided diagnostic (CAD) systems for lung disease detection utilizing X-ray images have been established over the last decade [14]. However, such systems fell short of the requisite effectiveness for lung disease identification and classification. The subsequent COVID-19-aided lung diseases have made such duties extremely difficult for such CAD systems. It is critical to notice the emergence of pneumonia in the lungs and classify it as bacterial, viral infection or COVID-19. Digital technology has recently grown in importance across the world. Machine learning [15,16,17,18] and deep learning [19] can be quite useful in this endeavor. Previously, Heyat et al. used medical machine learning to detect sleep disorders, mental stress, and female disorders based on signal and experimental data [20,21,22,23,24,25]. Transformer models [26] have lately exhibited outstanding performance on a wide variety of language tasks, including machine translation [27], question answering and text categorization. With its scalability to extremely high capacity models, Transformer models’ enormous significance has become obvious [28]. Transformer framework advances in the Natural Language Processing (NLP) sector have piqued the interest of computer vision researchers in adapting these models for visual and multi-modal learning challenges. However, because visual data has a consistent structure (e.g., temporal and spatial coherence), unique network architectures and training strategies are required. As a result, Transformer frameworks and modifications have been utilized effectively for object detection, image recognition, segmentation [8], image generation, image super-resolution, video comprehension, visual question answering, text-image synthesis, and several other tasks. Transformer architectures are built on a self-attention technique that learns the links between sequence parts. Even though attention models have been widely employed in both recurrent and feed-forward networks [29], transformers are purely based on the attention mechanism with a novel approach (i.e., multi-head attention) that is designed for parallel computation.

X-ray of chest Pneumonia detection has long been recognized as a challenge by academics [30]. In their network backbone, Albahli et al. [30] advocated employing Residual Blocks and dilated convolution in place of conventional convolution blocks, attaining a Recall rate of 96.7% and an F1_score of 92.7%. Elshennawy et al. [31] assessed the performance of four models, two of which were pre-trained models (MobileNetV2 and ResNet152V2), a CNN model built from scratch, and an LSTM model. The models were assessed with different parameters using standard classification assessment measures. Wang et al. [32] emphasized the need for early detection of pneumonia sickness. They used transfer learning and model adaptation methodologies to forecast the illness using the VGG-16 and Xception models, reaching detection accuracy of 87% and 82%, respectively, for the VGG-16 Xception models. Talo et al. [33] used the transfer learning approach to diagnose pneumonia illness using the ResNet152 model. Without any preprocessing or feature extraction, it identified 97.4% of the collection. Varshni et al. [34] investigated the diagnosis of Pneumonia using numerous models based on a convolutional neural network (CNN), which they used for extracting features via transfer learning and several classifiers as predictors. Their findings show that pre-trained CNN models mixed with supervised classifier models can aid in the evaluation of chest x-ray images, notably in the detection of Pneumonia. The authors also observed that using DenseNet-169 for feature extraction and SVM (Support Vector Machines) as the predictor produced the best results. In contrast to transfer learning-based efforts, Stephen et al. [35] employed data augmentation to construct a trained CNN for pneumonia diagnosis. The model’s effectiveness was tested with various image dimensions, with a 200 by 200 RGB image yielding the best results (93.73%). To classify chest X-ray pictures as normal, bacterial, or viral pneumonia. Hammoudi et al. [36] used numerous deep learning models (ResNet50, ResNet34, VGG-19, DenseNet169, and Inception ResNetV2—RNN). Sirazitdinov et al. [37] used RetinaNet and Mask R-CNN to detect lung pneumonia using a Chest X-ray image database, with a recall of 79.3%. Liang and Zheng [38] presented a transfer learning strategy for diagnosing pediatric pneumonia with a recall rate of 96.7% and an F1_score of 92.7%. The author also used the CNN and VGG16 models, achieving 90.5% accuracy, 89.1% precision, 96.7% recall, and 92.7% F1_score for the CNN model, respectively. Chouhan et al. [39] employed Guangzhou Women’s and Children’s Medical Center using a transfer learning algorithm with a 96.4% success rate. Siddiqi et al. [40] employed a sequential 18-layer CNN to identify pneumonia and achieved an accuracy of 93.75%, whereas Jain et al. [41] achieved 95.62% accuracy, 95% recall, and 96% precision for pneumonia diagnosis from chest X-ray images.

The authors of [42] investigated ResNet-50, ResNet34, MobileNet V2, GoogleNet, Inception V3 VGG16, SqueezeNet, and AlexNet models for early COVID-19 infection detection using CXr pictures. For the best model selection, parameters such as learning rate, number of epochs, and batch size were considered. The assessment findings revealed that the ResNet34 model outperformed all other assessed models, with an accuracy of 98.33 percent. Ozturk et al. [43] employed X-ray images to diagnose COVID-19 using CNN-based transfer learning (TL). The photos were put directly into the Inception-V3 model, which achieved 96% accuracy. Reference [44] authors produced a COVID-19 test model (VGG-16 and ResNet-50) based on the COVID-19 radiography dataset, with three classes: normal, COVID-19, and other pneumonia infection. The VGG-16 model fared the best, with a 97.67% accuracy. Furthermore, Das et al. [45] indicated that they improved COVID-19 detection performance utilizing CXR pictures by adjusting data augmentation and CNN model parameters. The VGG-19 and ResNet-50 models performed better as a result of this strategy. However, a suggested model called CovidXrayNet, which is built on EfficientNet-B0 and optimization, was presented, resulting in an of 95.82% when tested with data from two independent databases. Rajpal et al. [46] investigated the COVID-19 detection problem as a three-class classification problem: normal, COVID-19, and pneumonia. The proposed building was divided into three parts. ResNet-50 with TL was used in the first stage to generate 2048 parameters. The second portion employed Principle Component Analysis (PCA) to choose 64 characteristics from a total of 252. In the third module, the attributes obtained in the previous two parts were combined and classified, yielding a classification accuracy of 0.98%. SARS-Net was proposed by Kumar et al. [47] for COVID-19 identification using CXr. In that analysis, the open COVIDx database including CXr data was used. According to quantitative research, the proposed design has a higher accuracy of 97.60%. The authors of Reference [48] train and test a ResNet50 architecture with a small database of 50 COVID-19 examples from the Cohen et al. source and 50 normal cases from Kaggle, attaining 98% accuracy using re-sampling and five-fold cross-validation. In [49], TL was used to offer a novel framework called COVID-Net. The authors created a dataset comprising 8066 normal samples, 183 COVID-19 samples, and 5538 Pneumonia samples, while the test set included 100 pneumonia and normal samples, respectively, and 31 COVID-19 samples, yielding a 92% accuracy. The authors of [50] studied the COVID-19 variants using the transfer learning approach to tackle the New Stringency Indicators. We summarized our literature review in Table 1.

Nevertheless, even for professional and competent doctors, X-ray-based lung disease identification remains a mammoth task because X-ray images offer identical region information for various disorders such as pneumonia, COVID-19, and so on. As a result, traditional techniques of detecting lung disorders are time-consuming and energy-intensive, and it is difficult to employ a consistent methodology to establish which sort of lung disease a patient has. Many scholars have sought to improve CNN’s performance and have seen significant improvements over time. The CNN model, on the other hand, merely examines the connection between spatially nearby pixels in the receptive region defined by the filter size. As a result, identifying associations with distant pixels is challenging. As a result, this study proposed a Chest X-ray Image Based Feature Extraction Framework for accurate and fast lung disease identification. First, using fused fine-tuned pre-trained deep learning models, increased contour and correlations of lung disease-specific X-ray characteristics are retrieved. On the same hand, a global second-order pooling was applied for enhancing non-linear capabilities and taking advantage of comprehensive visual information across the fused pre-trained deep learning models. Furthermore, the Chest X-ray images are split into patches and positional embedding before passing them to the multi-head attention mechanism for robust global feature extraction. We utilized the Globalaverage2d layer, a Batch normalization layer, a dense layer with GeLu activation, another Batch normalization layer, and a dense layer with SoftMax’s activation for the classification Block. However, before selecting the suggested feature extractor models, this article first investigated numerous deep learning models using transfer learning. Furthermore, a thorough evaluation of the proposed model was conducted utilizing multiple datasets with multi-class classification (Normal, COVID-19, Pneumonia), and (Normal, COVID-19, Pneumonia, Lungs Opacity). The main contribution of this paper is summarized as follows:

- This research offers a refined Chest X-ray Image Based Feature Extraction Framework for Lung Disease identification that is significantly discriminative in identifying Pneumonia, COVID-19, and Lung Cancer Diseases.
- We offer explainability-driven, medically explainable visuals that emphasize the crucial regions relevant to the model’s prediction of the input image.
- We established a novel technique for improving ensemble models by using the integration of global second-order pooling and multi-head self-attention.
- This work examined many pre-trained deep learning models, providing a unique ensemble deep learning model that acts as the suggested model backbone, tackling the problem of the requirement for large-scale data.
- We reported a well robust deep learning method in Accuracy, Specificity, Sensitivity, Precision, F1 Score, Confusion matrix, and AUC using receiver operating characteristics (ROC) for detecting Pneumonia, COVID-19, and Lung Cancer Diseases based on a detailed experimental evaluation of the proposed model and comparison with state-of-the-art results.

This paper is structured as follows; Section 1 talks about the introduction and literature review of the study. Section 2 describes the material (Dataset) used while the methodology and model architecture are presented in Section 3. The attended result is presented in Section 4 alongside the experimental setup and result analysis. Section 5 presents the result discussion, ablation studies and result comparison with the state-of-the-art models.

## 2. Materials and Methods

This study implemented its idea in a vision transformer implementation style. Vision transformer [51] is an encoder-only based type of attention-based transformer [26] widely deployed in the Natural language processing (NLP) domain that has made it simpler for visual and pattern recognition domain in image data. In the absence of pre-image analysis tasks, such as image classification, the input image $x\in R^{H\times W\times C}$, is divided into N image patches, $x_{p}^{\left(i\right)}\in R^{H\times W\times C}$, where i∈{1,⋯N} and each patch has the form *P* × *P* in 2-D, *C* specifies the number of channels and $N=\frac{H\times W}{P\times P}$ The image patches are then employed successfully as a succession to the transformer’s input images. Patch embeddings are generated by flattening the input patches and then mapping them to a *D* dimensional latent vector using a learnable linear projection. In the series of patch embeddings $\left(Z_{0}^{0}=x_{class}\right)$, a trainable embedding is embedded. The class token’s last transformer layer state $Z_{L}^{0}$ contains the classification information that the model can obtain from the image in a concise way (y). During both pre-training and fine-tuning, the classification head is connected to $Z_{L}^{0}$. Standard learnable 1D position embeddings are added to the patch embeddings to maintain critical positional information. The encoder receives the final result sequence as input. The encoder is made up of alternating layers of multiheaded self-attention (MSA) and MLP blocks. Before each block, the layer norm (LN) is applied, followed by residual or skip connections. Additionally, we introduced a global second-order pooling [52] for utilizing comprehensive image information across a network in order to implement in an effective manner a higher-order interpretation of the output layers of the fused models for enhancing the non-linear function of the fused model before passing the features to the encoder. The global second-order pooling technique is utilized to create a transformation matrix from a 3D vector generated by the fused layer as an input, which is then used for vector multiplication along the entire continuum and spatial context.

### 2.1. Dataset

Some existing works use proprietary datasets to evaluate their approaches, while others mix data from many publicly available sources. Two huge publicly available datasets were used in this work, as stated below:

#### 2.1.1. Data_A

This dataset comprises medical CXr images for four distinct classes: Normal, Pneumonia, Lung Opacity, and COVID-19, which were gathered by researchers from Qatar University Doha Qatar, University of Dhaka, Bangladesh, as well as medical professionals and researchers from Pakistan and Malaysia. The COVID-19 Radiography Dataset [53] is titled. 3616 COVID-19 samples, 10,192 Normal samples, 6012 Lung Opacity samples, and 1345 Pneumonia samples make up the four classes. The images are in the png (Portable Network Graphics) file type and have a resolution of 299 × 299 pixels. Only 3000 images per class were sampled for training, 300 samples for validation, and 300 samples for testing in this paper. We executed a data augmentation using the Python Augmentor pipeline to obtain the number of samples needed for the experiment because the Pneumonia samples were fewer than 3000.

#### 2.1.2. Data_B

Badawi et al. [54] obtained the ChestX-ray-15k dataset from eleven distinct sources. With 3500 and 1500 images, respectively, this dataset has a balanced number of Chest X-ray images for training/validation and testing. Normal, COVID-19, and Pneumonia are the three distinct chest X-ray grades. All the images in this category are in portable network graphics (.png) format, albeit with varying spatial resolutions. The validation set was made up of 500 images from each class of the test set.

**Figure 1:**
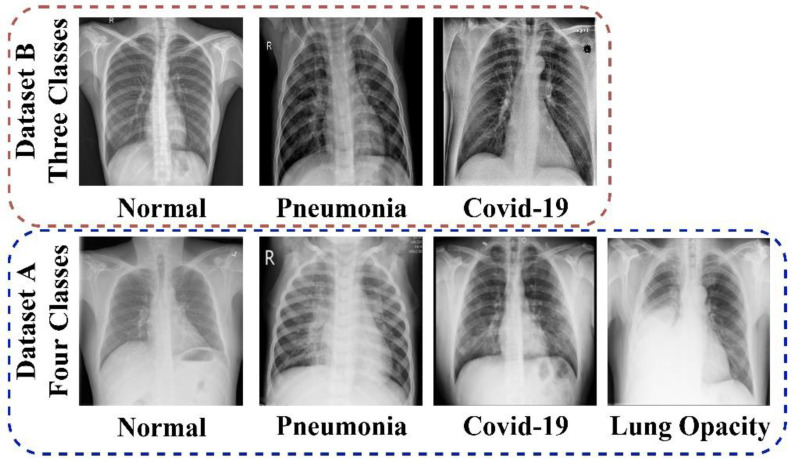
Sample of the employed dataset.

These datasets are used to solve the multi-class scarcity problem and conduct a multi-class prediction job for lung diseases. Bilinear interpolation was used to scale all the CXr images to 224 by 224 pixels. Meanwhile, the following data transformations were performed to increase the number of images per class: zoom range = 0.2, rotation range = 1, and horizontal flip = True. Figure 1 shows several examples of visual perspectives for each of the classes. Table 2 displays the distribution splits of the dataset by class. The training set, validation set, and testing set are all randomly selected from the dataset for each class.

### 2.2. Model Architecture

**Figure 2:**
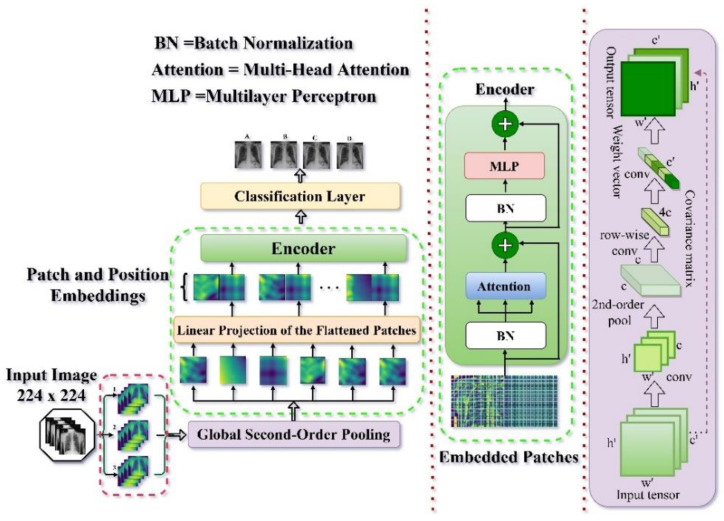
The proposed model organizational structure. The DenseNet201 (shown with 1), VGG16 (shown with 2), and GoogleNet architecture (shown with 3) serve as the network backbone to help in feature extraction. The fused features are passed via a global second-order pooling before being split into N patches and linear projection is employed to embed them. After adding position embedding, the sequence is supplied to an encoder, which then passes it to the classification/detection layer for prediction.

As shown in Figure 2, we present a patch-based Chest X-ray Image Feature Extraction Framework for Lung Disease Detection that is accurate and dependable. The captured features from the network backbone after passing through global second-order pooling were implemented in the encoder in two distinct layer configurations: a multi-head self-attention layer and an MLP layer. The shortcut connection and the normalizing layer are used to build each layer.

The output is as follows:

(1) $$x_{i+1}=f_{\mathrm{LN}}(x_{i}+f(x_{i})),$$

where *x_i_* symbolizes the layer *i* input and layer *i* − 1 output, *f*_LN_ symbolizes the normalization layer, *f*(·) symbolizes either the multi-head attention *f*_ATT_(·) or MLP *f*_FFN_(·).

**Figure 3:**
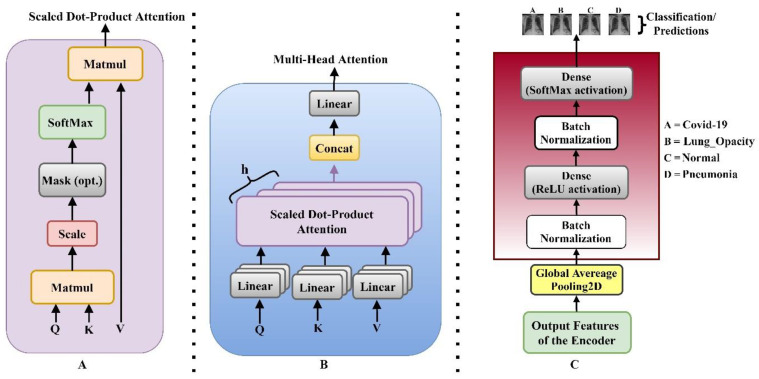
Illustrations of the implemented encoder. (**A**) Illustrates the Scaled dot-product attention (**B**) Multi-head Self-Attention network showing the several attention layers (Q, K, and V) running in parallel where (**C**) shows the implemented MLP block.

The multi-head self-attention layer, which is based on scaled dot-product attention as illustrated in Figure 3A, is utilized to capture the interdependence among input tokens. The Scaled dot-product attention algorithm aims to find important information from the source sequence for the target sequence. We infer the output of the scaled dot-product attention as shown in Equation (2). where n represents the length of the source and target sequences, m denotes the hidden dimension, and the target sequence is represented as $Q\in R^{n\times m}$, while the source sequence is represented as $K\in R^{n\times m}$ and $V\in R^{n\times m}$.

(2) $$f_{\mathrm{Scaled}\;\mathrm{Dot}-\mathrm{Product}\;\mathrm{Attention}}(Q,K,V)=softmax(\frac{QK^{T}}{\sqrt{m}})V$$

where the row-wise SoftMax is represented *softmax*(·). Because the output of SoftMax often has one dimension much bigger than the other dimensions in each row, one scaled dot-product attention attends just one place in each row (for each target token). Multi-head attention was used to attend to several places using multiple scaled dot-product attention simultaneously, as seen in Figure 3B and mathematically described as

(3) $$f_{\mathrm{ATT}}(Q,K,V)=[{\mathrm{head}}_{1},\cdots ,{\mathrm{head}}_{h}]W^{\left(O\right)},$$

where ${\mathrm{head}}_{i}=f_{\mathrm{Scaled}\;\mathrm{Dot}-\mathrm{Product}\;\mathrm{Attention}}(QW_{i}^{\left(Q\right)},KW_{i}^{\left(K\right)},VW_{i}^{\left(V\right)})$, h depicts the number of attention heads, *W*^(·)^ represent the learnable entities. The MLP layer configuration is of two MLP Block as seen in Equation (4);

(4) $$f_{\mathrm{FFN}}(x)=Ø(xW^{\left(1\right)})W^{\left(2\right)},$$

where the non-linear function is depicted as Ø(·) and *W*^(·)^ depicts parameters. After a Global Average Pooling 1D, the GeLU activation function was employed at the first layer, while the SoftMax activation function was utilized after Batch Normalization at the second layer, as shown in Figure 3C. Batch Normalization is the layer of a neural network that allows the following layers of the model to adjust more independently [55]. It’s used to scale the activations of the input layer and make the output of the preceding layers more realistic. Training becomes more successful when batch normalization is utilized, and it may also be used as a regularization to reduce model overfitting. The Gaussian Error Linear Unit (GeLu) activation is the initial Dense layer activation. Because of its deterministic nonlinearity, which includes a stochastic regularization effect that leads to a large performance boost in most models with intricate structures, the GeLu was used in this study. The fundamental function of the SoftMax layer is to transform the output information from the encoding layer into a probability interval (0, 1). In this work, the detection was treated as a multi-classification challenge. Following that, the input samples are passed to the encoding network, which then transfers their outputs into the probability interval (0, n) through the SoftMax layer, as seen below:

(5) $$l_{i}=P\left(t_{i}|S_{i}\right)=\frac{1}{1+e^{-\left(W_{c}u+b_{c}\right)}}\varepsilon \left(0,n\right),$$

where the weight matrix and the bias term are denoted as *W_c_* and *b_c_* respectively. Adam optimizers are used in this research. To compute the loss between the ground truth and the identified item, this study used a modified loss function categorical smooth loss and a categorically cross-entropy loss. The addition of smoothing the labels function to the cross-entropy loss function, as shown below, is known as categorical smooth loss;

(6) $$L\left(\theta \right)=-\frac{1}{N}\sum_{i=1}^{N}\left(y_{i}^{T}\log\left({\hat{y}}_{t}\right)+{\left(1-y_{t}\right)}^{T}\log\left(1-{\hat{y}}_{t}\right)+label_{smoothing}=n\right).$$

The extracted feature *i* from the backbone model is portrayed as *x_i_*, in Equation (1), and the attention layer configuration generates outputs as described in Equation (7) and the MLP layer configuration as given in Equation (8).

(7) $$x_{2i+1}=f_{\mathrm{LN}}\left(x_{2i}+f_{\mathrm{ATT}}\left(x_{2i},x_{2i},x_{2i}\right)\right)$$

(8) $$x_{2i+2}=f_{\mathrm{LN}}\left(x_{2i+1}+f_{\mathrm{FFN}}\left(x_{2i+1}\right)\right).$$

The attention layer uses Equation (2) to establish Q, K, and V values = *x*_2*i*_, capturing the reliance between tokens within the same sequence, also known as self-attention.

### 2.3. Feature Extraction

**Figure 4:**
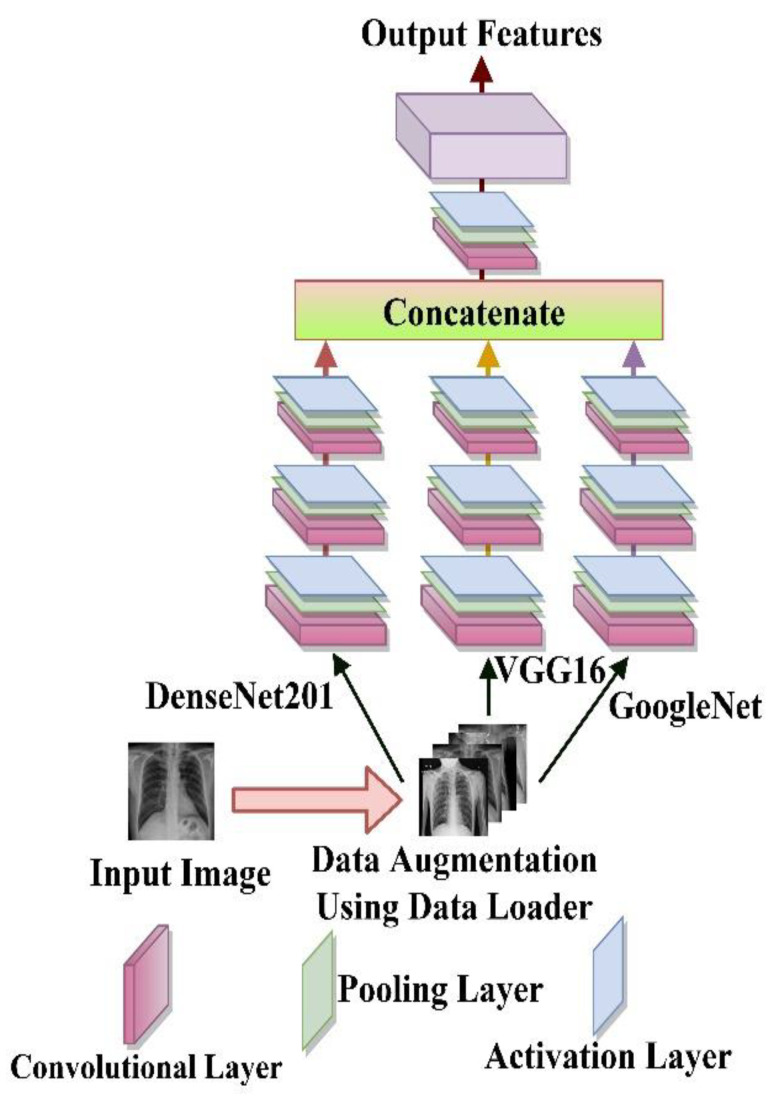
Mode of Feature extraction of the proposed study. From the network backbone up to the global second-order pooling layer.

As illustrated in Figure 4, this work combines deep features collected from DenseNet [56,57], VGG16 [58], and GoogleNet [59] using Ensembling algorithms [60,61]. DenseNet [41] architecture is a classification model that involves connecting layers in a feed-forward manner (with identical feature-map size), this design ensures knowledge transfer across network tiers. The output of the previous layer and the output of the following layer is concatenated (.). VGG16 [42], a deep learning architecture first preprocess its input data before being input into a tiered convolutional layer with three susceptible filters and a constant stride of one. Spatial pooling is then carried out using five max-pooling convolutional layers with a 2 × 2 filter and stride of 2. Two fully connected layers (FC) and SoftMax activations at the end of the design make up the model structure. GoogleNet [59] uses inception modules, which enable the model to select between several convolutional hyperparameters per block and are intended for image classification and identification. It consists of 22-layers. By using an inception module as the first layer, which is then piled upon itself, GoogleNet seeks to increase the computational complexity of basic CNN by applying parallel filtering on the input from the previous layer.

Ensembling is the process of combining different learning algorithms to improve the overall performance of current models by combining many models into a single trustworthy model. The fusion is calculated as follows:

(9) $$F_{Pre-trained}=\left\{f_{DenseNet},f_{VGG16},f_{GoogleNet}\cdots ,f_{1\;x\;n}\right\},$$

where *n* is the number of pre-trained models that have been chosen. The features are then concatenated into a single vector, as shown below:

(10) $$F_{Ensemble}=\sum_{i=1}^{3}\left\{f_{Pre-trained}\right\}.\;$$

*F_Ensemble_* is then run through a 2D convolutional layer with a kernel size of 1, padding = ‘same’, and activation = “ReLU.” Immediately comes the Global second-order pooling which is intended to use the comprehensive image information across the network for an effective higher-order interpretation of the output layers of the fused models thus enhancing the non-linear function of the fused model. Zeropadding2D was used to zero-pad the output of the new layer (padding = ((0, 5), (0, 5)).

(11) $$Backbone=F_{Ensemble}+2D_{Conv.Layer}+Global\;second-order\;pool+Zeropadding.$$

### 2.4. Evaluation Metrics

The robustness of the suggested model was assessed using a variety of evaluation indicators. Accuracy, precision, specificity, F1_score, sensitivity, and area under a receiver operating characteristic curve are among the measurements [62,63,64,65,66,67,68]. TP stands for True Positive, FP for False Positive, TN for True Negative, and FN for False Negative. The following are the metrics we used:

(12) $$Accuracy=\frac{TP+TN}{\left(TP+TN\right)+\left(FP+FN\right)}*100.$$

(13) $$Precision=\frac{TP}{TP+FP}*100.$$

(14) $$Specificity=\frac{TN}{N}*100=\frac{TN}{TN+FP}*100.$$

(15) $$Sensitivity=\frac{TP}{P}*100=\frac{TP}{TP+FN}*100.$$

(16) $$F_{1}\;score={\left(\frac{SEN^{-1}+PRC^{-1}}{2}\right)}^{-1}=\frac{2*TP}{2*TP+FP+FN}$$

The AUC measures a classifier’s performance, while the probability curve gotten from plotting at different threshold settings, the FP rate is referred to as the ROC (Receiver Operating Characteristic). The AUC indicates how well the model distinguishes between the different lung disease instances. The higher the AUC, the better.

## 3. Results

The many experiments carried out in this study are explained in this section. First, the experiment was carried out with pre-trained models, which include dual learning rates and loss functions.

### 3.1. Experimental Setup

All experiments have been performed on a Desktop Computer with 64.0GB RAM and an NVIDIA GEFORCE RTX-3080 Ti 10 GB graphics processing unit with an Intel(R) Core (TM) i9-10850K CPU running at 3.60 GHz (GPU). For the implementation, this research used the open-source Keras framework and TensorFlow. During the training phase, the suggested deep learning models were fine-tuned and assisted using the same training and testing settings and methodologies. The es callback early-stopping approach with the patience of 10 was also evaluated. An es callback is a component that may perform operations at different stages of learning, such as at different batch intervals, epoch intervals, and so forth. The Adam optimizer is used for hyper-parameter optimization, with a clip value of 0.2 and an epoch of 100. The encoder uses eight heads with a patch size of 2 and a drop rate of 0.01 for all layers. Meanwhile, the shift size is calculated using embed dim of 64 (embed dim indicates the dimension by which high-dimensional vectors are converted to low-dimensional vectors without loss), num_MLP of 256 (this indicates the number of multi-linear perceptron’s), a window size of 2, and global average pooling (GAP). The hyperparameters utilized in the studies are listed in Table 3.

### 3.2. Classification Results

The classification findings of the various methodologies used in this work are discussed in this section. Because the backbone is made up of unified deep learning models, we’ll start with their findings, which we’ll show using the loss functions and learning rate we used.

#### 3.2.1. Backbone Model Selection

**Figure 5:**
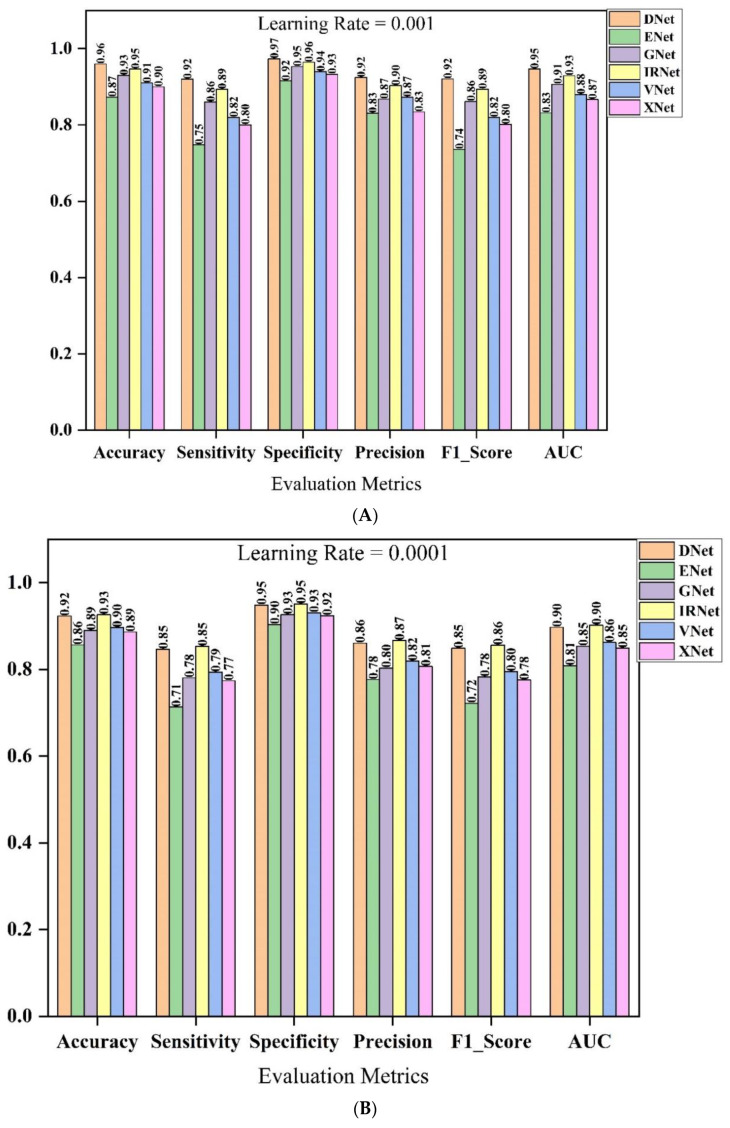
Classification performance result of the pre-trained models for the backbone selection using the Data_A. (**A**) Pre-trained model selection using a learning rate of 10^−4^ and (**B**) Pre-trained model selection using a learning rate of 10^−3^. DNet stands for DenseNet201, ENet stands for EfficientNetB7, GNet stands for GoogleNet, IRNet stands for InceptionResNetV2, VNet stands for VGG16 and XNet stands for Xception, respectively.

Six pre-trained deep learning models were identified during the selection of the implemented backbone, namely DenseNet201, VGG16, GoogleNet, InceptionResNetV2, Xception, and EfficientNet network architecture. Table 4 shows the outcomes of the pre-trained deep learning models that were used, as shown visually in Figure 5. During the backbone model selection experiment, Data_A was employed.

In terms of the assessment measures employed in this study, the DenseNet model achieved the best result. It outperforms the other models in terms of employed learning rates. The InceptionResNetv2 architecture comes after the DenseNet architecture, before the GoogleNet and VGG16 models. The Sensitivity, Specificity, F1 score, and AUC score were the most important metrics to consider while choosing feature extractors. The more precise the model’s categorization and prediction are, the better the outcomes of the chosen assessment metrics. With a learning rate of 10^−4^, the DenseNet achieved 0.91981% sensitivity, 0.97325% specificity, 0.92088% F1_score, and 0.94651% AUC. The recorded results for the InceptionResNetV2, GoogleNet, and VGG16 architectures are: 0.89385% sensitivity, 0.96434% specificity, 0.89398% F1_score, 0.9291% AUC score, 0.86024% sensitivity, 0.95322% specificity, 0.86188% F1_score, 0.90673% AUC score, 0.81988% sensitivity, 0.93985% specificity, 0.82008%. The pre-trained models outperformed Adam optimizers with a learning rate of 10^−4^ when compared to Adam optimizers with a learning rate of 10^−3^.

Table 5 shows the ROC while Table 6 shows the PR curve performance of the network backbone selection. The objective of this is to see how well the models do in their respective classes. The DenseNet design had the greatest COVID-19 ROC class performance, with an area of 0.95583 percent, followed by the GoogleNet architecture with an area of 0.90296%. The Xception and the EfficientNet are followed by the InceptionResNetV2 with an area of 0.86819%, VGG16 with an area of 0.85526%, and finally the InceptionResNetV2 with an area of 0.86819%. While compared to the GoogleNet architecture, the InceptionResNetV2 COVID-19 class had a superior area when utilizing the Adam optimizer with a learning rate of 10^−3^. We also analyzed the computational cost of all six models, i.e., the amount of trainable and untrainable parameters of the architecture, to complete our backbone network choices. As a result, we concluded that the feature extractors should be fused using DenseNet, GoogleNet, and VGG16.

#### 3.2.2. Classification Results Using Data_A

Table 7 shows the classification performance of the proposed model and the backbone network. Two distinct learning rates and loss functions were used in the studies. In both implemented loss functions, the results obtained with a learning rate of 10^−3^ surpass those obtained with a learning rate of 10^−4^. Despite this, the model had a lower performance when using the learning rate of 10^−3^ and categorical smooth loss, with an accuracy of 0.96667%, Sensitivity of 0.93314%, Specificity of 0.97772%, Precision of 0.93895%, F1_score of 0.93391%, and AUC area of 0.95543 percent compared to using the categorical cross-entropy loss, which had an accuracy of 0.98%, Sensitivity of 0.94965%, Specificity of 0.98992%, Precision of 0.95508%, F1_score of 0.95216% and AUC area of 0.96976%. The Adam optimizer is favored in all other situations with a learning rate of 10^−3^ and categorical cross-entropy.

Table 8 explain how the Receiver Operating Characteristic (ROC) and Table 9; Precision-Recall (PR) are used to confirm these findings. To evaluate the precise prediction rate of the classes Normal, COVID-19, Pneumonia, and Lung Opacity, the ROC and PR curves are employed. However, the hyperparameters had a significant impact on the accurate prediction rate of the models, as the learning rate of 10^−4^ with categorical cross-entropy surpassing the learning rate of 10^−3^ with categorical smooth loss. Table 6 shows that the Adam ROC class performance was 0.95606% for COVID-19, 0.98206% for Lung Opacity, 0.95559% for Normal, 0.92801% for Pneumonia, and 0.92% for AP, 0.95% for Lung Opacity, 0.83% for Normal, 0.87% for Pneumonia. When comparing the four classes, the COVID-19 class outperformed the lung opacity class in the majority of the optimum settings in terms of ROC and AP regions.

**Figure 6:**
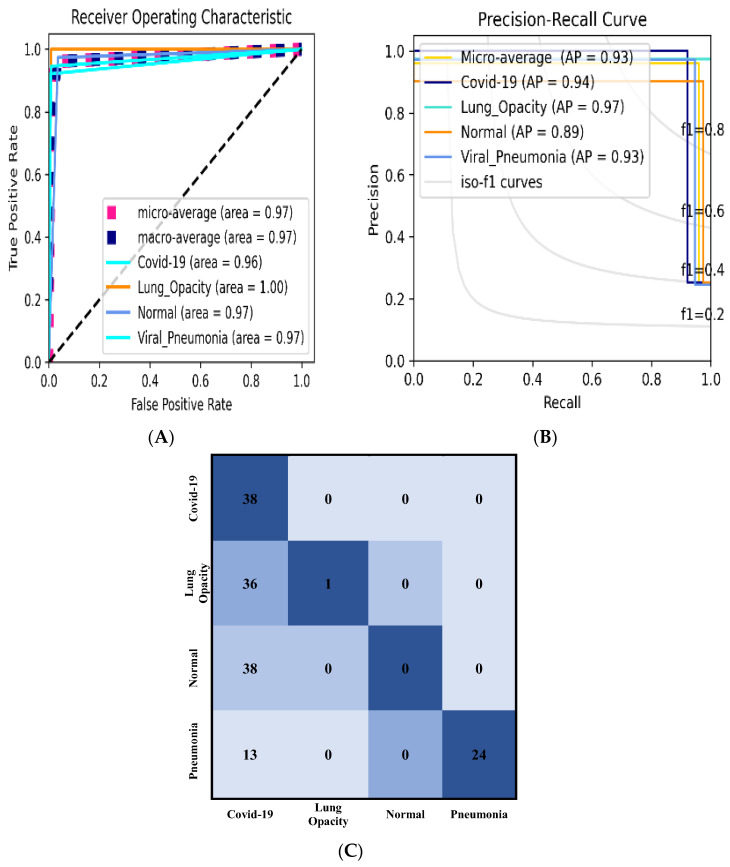
The optimized setting results include (**A**) ROC and (**B**) PR curve of the 10^−4^ learning rate and categorical cross-entropy loss function, and (**C**) Hit rate diagram, based on Data_A.

The ROC curve and the AP Curve, as mentioned in Table 7, are graphically shown in Figure 6. The HIT Rate data were utilized to further assess the model, as illustrated in Figure 6. When the adjustments are done, the “Hit Rate” is calculated by dividing the full sequence number (obtained by removing the number of Targets plus Mistakes); the “Miss Rate” is 1 minus the “Hit Rate.” COVID-19 had a 38 percent hit rate. As the Normal class hit rate recorded 38 versus the 10^−3^ learning rate and categorical smooth loss function, the performance of the 10^−4^ learning rate and categorical cross-entropy loss function are chosen.

#### 3.2.3. Classification Results Using Data_B

The proposed model’s classification results on data B are shown in this section. Unlike the experimental analysis using data A, this paper only employed the Learning rate of 10^−4^ and the categorical smooth loss function in this article.

Table 10 shows the obtained result in terms of the assessment measures used. Table 8 shows that the suggested model performed significantly better in classification, with overall accuracies of 98.19 percent, sensitivity of 97.29%, specificity of 98.64%, precision of 97.29%, F1_score of 97.29%, and AUC of 98.10%. This demonstrates how the backbone model performs better when combined with the proposed model. The qualitative evaluation results in terms of ROC and PR curves, on the other hand, showed similar results. The COVID-19 samples were predicted more accurately than the other two classes in terms of the ROC and precision-recall curves, with the pneumonia class recording an AUC of 98.42% and the AP being more significant than the viral pneumonia class with an average of 96.0%. COVID-19 had an AP of 97.96%, while Pneumonia had an AP of 97.85%. The evaluation results in the normal class were attained with a little lower AUC and even PR rates of 97% and 96.06%, respectively. This was owing to the well-known random deep-learning procedure for fine-tuning the trainable parameters.

**Figure 7:**
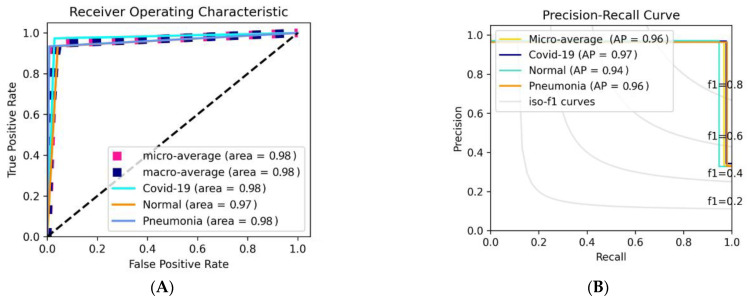

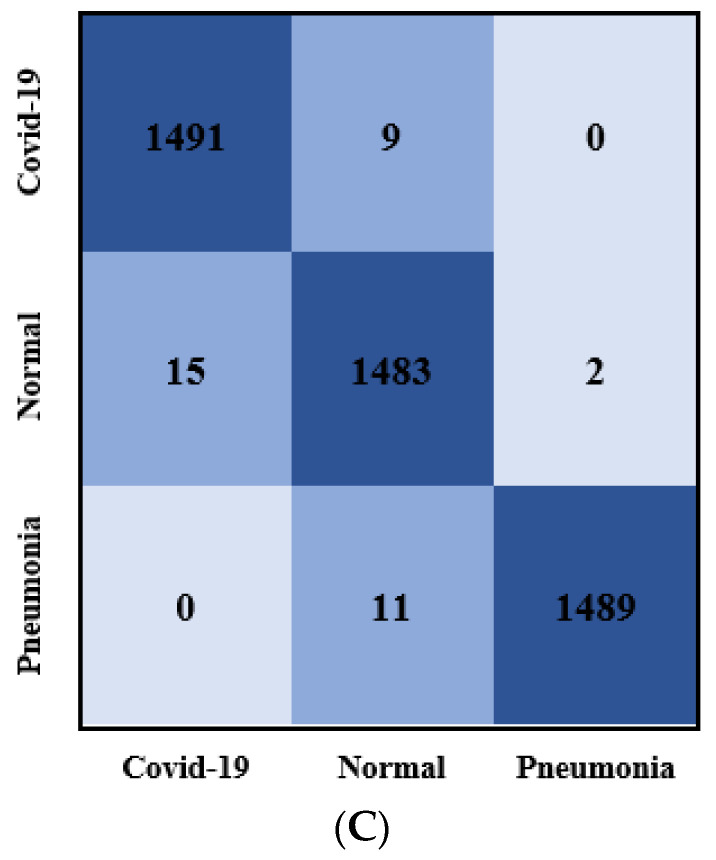
The experimental results include (**A**) ROC and (**B**) PR curve, and (**C**) Confusion Metrics, based on Data_B.

The Diagrammatic representation of the ROC curve and the AP Curve recorded in Table 8 and Table 9 for the Data_B experiment is shown in Figure 7A,B. To further elaborate on the performance of the proposed model on Data_B, we used the confusion metric instead of the Hit Rate as used in Data_A since the testing set of Data_B is much bigger in terms of the number of samples. Figure 7C shows the confusion metrics score of Data_B. From the diagram, the proposed model predicted 1491 samples of COVID-19 Class correctly, while misclassifying 9 samples to be Normal class. 1483 Normal class samples were predicted correctly while 15 samples were misclassified as COVID-19 samples and 2 samples as Pneumonia samples. For the Pneumonia Class, 1489 samples were correct while misclassifying 11 samples were Normal samples.

## 4. Discussion

The results of this experiment show that the proposed model for lung disease diagnosis is quite accurate. The results are described using the hyperparameters that were used in this investigation. We look at how the specified loss function affects categorical cross-entropy loss and the advantage of the 10^−4^ learning rate over the 10^−3^ learning rate. When using a learning rate of 10^−3^ and a categorical cross-entropy Loss Function as an assessment criterion, the Adam optimizer performs significantly better. The categorical cross-entropy yielded a better result than the categorical smooth loss in most cases. However, as compared to other loss functions, label smoothing appears to aid the model in detecting the damaged area, according to various papers. The proposed model in this paper makes use of patches and positional embedding, allowing the model to focus on all the damaged areas in patches while keeping the potions in mind for rebuilding. The model’s performance was boosted by the feature extraction strategy, which paid special attention to global features. According to the Data_B, using soft targets that are a weighted average of the hard targets and uniform distribution over labels, the label smoothing loss function technique aids in the generalization and learning speed of a multi-class neural network being regularly and significantly enhanced. This label smoothing stops the network from being overconfident. However, we can see that the learning rate has an impact on the effect of label smoothing, as the suggested model label smoothing with a learning rate of 10^−3^ exceeds the learning rate of 10^−4^ in Data_A result. Before adding more complicated architectures to the networks, this study emphasizes the relevance of deep learning model feature extraction and hyperparameter adjustment in processing new data. The outcomes of this work could be useful for quickly deploying accessible AI models for the rapid, accurate, and cost-effective detection of COVID-19 infection.

### 4.1. Ablation Studies of the Proposed Model

The heat maps that describe the deep learning outcomes are presented in this section. In this study, the attention approach aids the model in highlighting the relevant features of the Chest X-ray images, resulting in the suggested model’s prediction capacity. The internal working structure of the Proposed model starts with the input image being divided into patches before adding the positional embedding. By merging the pixel layers in a patch and then stretching it to the proper input dimension, each patch is compressed into a vector representation. positional embedding demonstrates how the model interprets distance within the input image in terms of position embedding comparability, i.e., relatively close patches have a lot of position similar embeddings. For accurate feature extraction, patches and learnable embeddings are employed to treat each patch separately.

**Figure 8:**
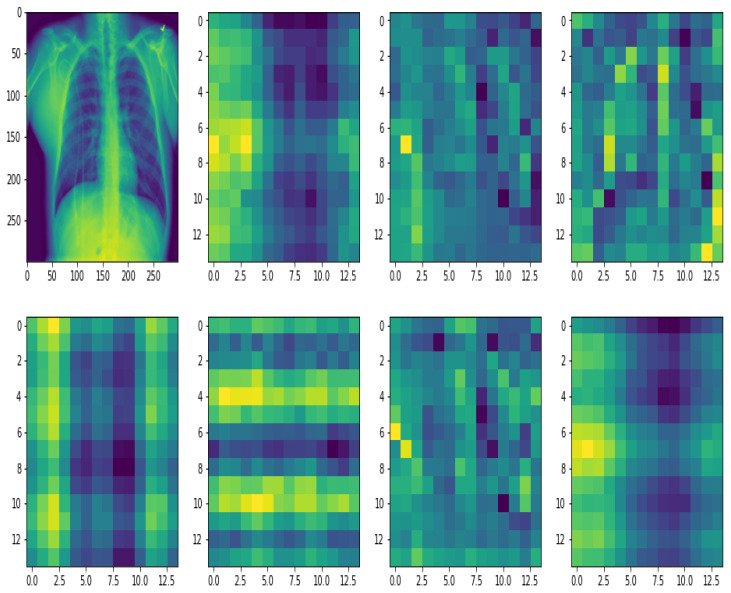
The proposed model focuses on visual features of the input image that are semantic information important for classification.

**Figure 9:**
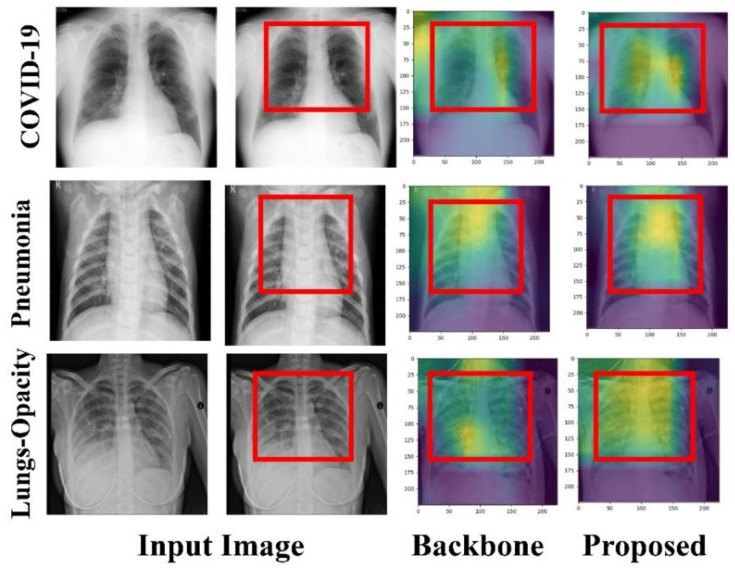
A Grad-CAM-based visualization of the proposed model on the different input data samples. The proposed model focuses on visual features of the image that are semantic information important for classification.

The model can remember where each patch was during the initial input and output thanks to positional embedding. To begin, 2D learnable convolutions are used to convert the patches. Figure 8 and Figure 9 confirm the suggested approach’s efficacy in boosting prospective ROIs, allowing the proposed model to focus on these regions rapidly and successfully and detect the disease, by examining the effects of the patch and embedding combo. It shows how the suggested model can generalize across the input frame, even within the simplest layers, thanks to the Self-attention heads. According to the diagram, the total distance in input images across which relevant data is assimilated is comparable to the receptive scale factor in CNNs and is highly recognized in our model due to our network backbone, which is an ensemble of pre-trained models, and thus we observed small attention scales in the small layers continuously. The attention heads focus on the bulk of the image in the lowest layers when the suggested model is implemented without a network backbone, i.e., by building features from scratch, implying that the model’s ability to compress information globally is used. The suggested model, as illustrated in Figure 8 and Figure 9, focuses on visual aspects that are semantic information that is vital for classification.

### 4.2. Comparison with the State-of-the-Art Based on Deep Learning Models

We compute and show the Accuracy, Precision, Sensitivity, and F1 score to compare the proposed model classification performance to existing cutting-edge approaches. When compared to other state-of-the-art approaches, the proposed model achieves the best overall accuracy of 98% (Table 11). For COVID-19 multiclassification, Wang et al. [53] suggested using COVIDNet, but Khan et al. [69] suggested using CoroNet. The COVIDNet model, on the other hand, beats the CoroNet model, with an accuracy of 90.78%, precision of 91.1%, an F1_score of 90.81% vs. 89.6%, the precision of 90.0%, and F1_score of 89.8%. Nonetheless, in terms of sensitivity, the CoroNet model outperformed the COVIDNet model recording 96.4%. The Mag-SD model was recommended by Li et al. [70], who attained 92.35% accuracy, 92.50% precision, 92.20% sensitivity, and 92.34% F1_score. To increase feature extraction comprehension of CXr images, Mondal et al. [71] and Shi et al. [72] advocated adopting an attention mechanism. Teacher-Student Attention was presented by Shi et al. [72]. The accuracy was 91.38%, which was better than the previous methods. The Local-Global Attention Network was introduced by Mondal et al. [71], and it surpassed earlier state-of-the-art models in terms of classification accuracy (95.87%), precision (95.56%), sensitivity (95.99%), and F1_score (95.74%). The author of Reference [73] used two different CXr classification algorithms with the same dataset in this study. With 92% accuracy, 91.75% precision, 94.50% sensitivity, and 92.75% F1_score, EfficientNetB1 (Strategy 2) produced the best classification results. Furthermore, the proposed technique has the maximum precision for COVID-19 circumstances, which means that a COVID-19 negative sample is rarely misidentified as a positive sample by the proposed classifier. Furthermore, the proposed approach has the highest recall score, indicating that the classifier can correctly identify the majority of positive COVID-19 samples. When compared to the baseline approaches, the suggested method has the highest F1_score, indicating that it is the most balanced in terms of precision and sensitivity.

This research leverages the Dataset_B classification performance as given in Table 12 to further test the proposed model’s superiority over state-of-the-art models. This comparison is based primarily on the many models used to detect pneumonia from chest X-ray images. Researchers have utilized several methodologies, such as the pre-trained model approach, ensemble model approach, and from-scratch model approach, as shown in the Table. Naralasetti et al. [74] used Deep CNN architecture and achieved a 91% accuracy rate. Ensemble models allow for a deeper understanding of the task and better results. However, when compared to the CNN model used by Dokur et al. [75], the proposed ensemble model fared poorly. In Accuracy, Precision, Recall, and F1_score, the CNN model outperformed the ensemble model by a factor of 3. For pneumonia detection feature extraction, Hammoudi et al. [36] implemented several deep-learning models. DenseNet121, VGG16, VGG19, and ResNet50 were the best of the implemented models. For X-ray pneumonia classification tasks, traditional models such as K-Nearest Neighbor (KNN), Nave Bayes (NB), Support Vector Machine (SVM), and Random Forest were used (RF). The authors concluded that DenseNet-169 when combined with ideal SVM RBF kernel hyper-parameter values, outperformed all other models tested. The from-scratch techniques were employed [76,77]. The researchers developed a novel model for pneumonia detection; however, the model’s performance was poor, with accuracy, precision, recall, and F1_score all falling below 90%. The authors [78] used the AlexNet architecture via transfer learning and produced the best classification accuracy among the state-of-the-art models with a 97.40% accuracy after analyzing the transfer learning methodologies. With a percentage accuracy of 98.19, precision of 97.29%, recall of 97.29%, and F1_score of 97.29%, the proposed model, which integrates all the investigated strategies, has proved to outperform all previous techniques.

### 4.3. Limitations and Future Works

The implemented model, on the other hand, has some limitations. To begin, the model was only investigated using chest X-ray scans, hence our findings are limited to chest X-ray images. There are various medical image modalities for lung disease detection and classification, including magnetic resonance imaging (MRI), ultrasound, and computed tomography (CT). In the future, the proposed approach will be applied to the listed medical image modalities. Furthermore, no Image Feature improvement procedures were examined in this investigation, and the degree of the lung disease (mild, moderate, or severe disease) was not considered. We also notice that the chest X-ray dataset only shows one series per patient, which supports [79]‘s thesis that a small dataset (one chest x-ray series per patient) cannot be utilized to predict whether a patient would develop a radiographic abnormality as the disease progresses. This will be thoroughly investigated in our upcoming investigation. Finally, the suggested model can be utilized to predict oral cancer, skin cancer, breast cancer, and other types of cancer.

## 5. Conclusions

This research focuses primarily on the identification of pneumonia and COVID-19, as these are the two most common lung diseases now afflicting people around the world. Lung disease identification was and continues to be an important part of epidemic diagnosis, and effective CXr data extraction aids in the correct diagnosis of lung illnesses, allowing for early detection and treatment. We present a unique Chest X-ray Image Based Feature Extraction Framework that split the images into patches and positional embeddings for accurate and fast lung disease identification. This paper first looked into the efficiency of six pre-trained deep learning models. Secondly, we proposed our model first the first step is to use a fusion model to extract deep features (generic features. Since the fusion model involves three concatenated models, there is a need for a higher-order representation of the features hence we introduced the global second-order pooling before the application of the multi-head self-attention network to analyze the input image regional features which in return pass the extracted features to the MLP layer for accurate lung disease classification and detection. To test the efficacy of the proposed approach, two publicly available datasets were employed. Data_A had a precision of 96.20% and an accuracy of 98.00%, while Data_B had a precision of 97.29% and an accuracy of 98.19%. We also assess the proposed model’s forecasting accuracy using an explainability-driven heatmap visualization to emphasize the key aspects influencing the prediction decision it makes. Not only are these decipherable visual clues a step closer to understandable AI, but they may also benefit professional radiologists in diagnosis. We have empirically proved the efficacy of the suggested strategy over state-of-the-art CNN-based algorithms in terms of precision, recall, and F1 score.

## Figures and Tables

**Table 1:** Summary of the related works covering the author, the proposed architecture, the type of lung disease they tackled and the achieved results.

Ref.	Architecture	Type	Result
[34]	Residual Blocks and Dilated Convolution	Pneumonia detection	Recall = 96.7% F1_score = 92.7%
[32]	Transfer Learning Via VGG-16 And Xception Models		Accuracy = 87%(VGG) and 82% (Xception)
[33]	Transfer Learning Via Resnet152 Model		Accuracy = 97.4%
[35]	Convolutional Neural Network (CNN)		Accuracy = 93.73%.
[37]	RetinaNet And Mask R-CNN		Accuracy = 79.3%
[38]	Transfer Learning		Recall Rate = 96.7% F1_score = 92.7%.
	VGG16		Accuracy = 90.5%, Precision = 89.1%, Recall = 96.7%, and F1_score = 92.7%
[39]	Transfer Learning		Accuracy = 96.4%
[40]	18-Layer CNN		Accuracy = 93.75%
[41]	CNN-Based Transfer Learning		Accuracy = 95.62%, Recall = 95%, and Precision = 96%
[42]	Resnet34	COVID-19	Accuracy = 98.33%
[43]	CNN-Based Transfer Learning		Accuracy = 96%
[44]	VGG-16		Accuracy = 97.67%
[45]	COVIDXrayNet		Accuracy = 95.82%
[46]	Resnet-50 With TL+ PCA + Ensemble		Accuracy = 98%
[47]	SARS-Net		Accuracy = 97.60%
[48]	ResNet50		Accuracy = 98%
[49]	COVID-Net		Accuracy = 92%

**Table 2:** Dataset distribution of the proposed method.

	Partition	Normal	Pneumonia	COVID-19	Lung Opacity	T_otal_	Total
Data_A	Training	3000	3000	3000	3000	12,000	14,400
	Validation	300	300	300	300	1200	
	Testing	300	300	300	300	1200	
Data_B	Training	3500	3500	3500		10,500	
	Validation	500	500	500		1500	1500
	Testing	1000	1000	1000		3000	

**Table 3:** Experiment hyperparameters optimization and settings.

Loss Function	Categorical Smooth Loss, Categorical Cross-Entropy
Optimizers	Adam
Learning rate	0.0001/0.001
Batch size	8
Reduce Learning Rate	0.2
Epsilon	0.001
Patience	10
Verbose	1
Es-Callback (Patience)	10
Clip Value	0.2
Epoch	100
Patch Size	(2, 2)
Drop Rate	0.01
Number of Heads	8
Embed_dim	64
Num_MLP	256
Window Size	Window Size//2
Input Size	(224 × 224)

**Table 4:** Selection classification results of the Backbone model using Data_A.

Models	Accuracy (%)	Sensitivity (%)	Specificity (%)	Precision (%)	F1 Score	AUC
Learning Rate: 10^−4^						
DenseNet201	0.9600	0.91981	0.97325	0.92453	0.92088	0.94651
EfficientNetB7	0.87333	0.74787	0.91542	0.83094	0.73639	0.83164
GoogleNet	0.9300	0.86024	0.95322	0.86795	0.86188	0.90673
InceptResNetV2	0.94667	0.89385	0.96434	0.90298	0.89398	0.9291
VGG16	0.9100	0.81988	0.93985	0.87301	0.82008	0.87986
Xception	0.9000	0.80032	0.93315	0.83420	0.80144	0.86674
Learning Rate: 10^−3^						
DenseNet201	0.92333	0.84691	0.94880	0.86073	0.84883	0.8976
EfficientNetB7	0.85667	0.71337	0.90408	0.77687	0.72241	0.80872
GoogleNet	0.89000	0.78094	0.92666	0.80353	0.78311	0.8538
InceptResNetV2	0.92667	0.85331	0.95097	0.86683	0.85618	0.90214
VGG16	0.89667	0.79374	0.93100	0.81953	0.79527	0.86237
Xception	0.88667	0.77400	0.92428	0.80688	0.77546	0.84914

**Table 5:** ROC and precision-recall curve of the backbone model using Data_A.

ROC (Area)	Macro-Average	Micro-Average	COVID-19	Lung Opacity	Normal	Pneumonia
Learning Rate: 10^−4^						
DenseNet201	0.95	0.95	0.95583	0.95061	0.93374	0.94595
EfficientNetB7	0.78	0.78	0.69737	0.93339	0.84445	0.85135
GoogleNet	0.91	0.91	0.90296	0.93734	0.86772	0.91892
InceptResNetV2	0.93	0.93	0.86819	0.97321	0.92904	0.94595
VGG16	0.88	0.88	0.85526	0.97345	0.87993	0.81081
Xception	0.87	0.87	0.78477	0.90588	0.8844	0.89189
Learning Rate: 10^−3^						
DenseNet201	0.90	0.90	0.86372	0.91497	0.8938	0.91892
EfficientNetB7	0.81	0.81	0.73167	0.87862	0.80028	0.82432
GoogleNet	0.85	0.85	0.80663	0.87515	0.82801	0.90541
InceptResNetV2	0.90	0.90	0.89427	0.92825	0.88064	0.90541
VGG16	0.86	0.86	0.81978	0.91521	0.84962	0.86486
Xception	0.85	0.85	0.78031	0.91964	0.83177	0.86486

**Table 6:** Precision-recall curve of the backbone model using Data_A.

Precision-Recall (AP)	Micro-Average	COVID-19	Lung Opacity	Normal	Pneumonia
Learning Rate: 10^−4^					
DenseNet201	0.87	0.87	0.89	0.81	0.92
EfficientNetB7	0.62	0.55	0.74	0.55	0.78
GoogleNet	0.77	0.75	0.82	0.68	0.88
InceptResNetV2	0.82	0.75	0.90	0.75	0.92
VGG16	0.72	0.78	0.86	0.61	0.71
Xception	0.69	0.62	0.76	0.62	0.84
Learning Rate: 10^−3^					
DenseNet201	0.76	0.73	0.76	0.69	0.88
EfficientNetB7	0.58	0.49	0.76	0.49	0.74
GoogleNet	0.66	0.63	0.63	0.60	0.86
InceptResNetV2	0.76	0.75	0.82	0.67	0.86
VGG16	0.68	0.65	0.73	0.60	0.80
Xception	0.65	0.60	0.75	0.56	0.80

**Table 7:** Classification results of the backbone vs. proposed model using Data_A.

Model	Accuracy (%)	Sensitivity (%)	Specificity (%)	Precision (%)	F1 Score	AUC
Learning Rate: 10^−4^, Loss Function: categorical_smooth_loss						
Backbone	0.95333	0.90701	0.96891	0.91419	0.90736	0.93796
Proposed Method	0.96000	0.91945	0.97325	0.92783	0.91934	0.94635
Learning Rate: 10^−3^, Loss Function: categorical_smooth_loss						
Backbone	0.94000	0.87909	0.95986	0.89431	0.88056	0.91948
Proposed Method	0.96667	0.93314	0.97772	0.93895	0.93391	0.95543
Learning Rate: 10^−4^, Loss Function: categorical cross-entropy						
Backbone	0.96333	0.92656	0.97551	0.93172	0.92677	0.95104
Proposed Method	0.98000	0.96017	0.98665	0.96209	0.9603	0.97341
Learning Rate: 10^−3^, Loss Function: categorical cross-entropy						
Backbone	0.83667	0.67372	0.89098	0.78304	0.65383	0.78235
Proposed Method	0.98000	0.94965	0.98992	0.95508	0.95216	0.96976

**Table 8:** Classification results of the backbone vs. proposed model based on ROC and PR curve using Data_A.

ROC (Area)	Macro-Average	Micro-Average	COVID-19	Lung Opacity	Normal	Pneumonia
Optimizer: Adaptive Moment Estimation, Learning Rate: 10^−4^, Loss Function: categorical_smooth_loss						
Backbone	0.94	0.94	0.94737	0.96903	0.90742	0.92801
Proposed Method	0.95	0.95	0.99107	0.97764	0.92481	0.89189
Learning Rate: 10^−3^, Loss Function: categorical_smooth_loss						
Backbone	0.92	0.92	0.94690	0.89656	0.92904	0.90541
Proposed Method	0.96	0.96	0.95606	0.98206	0.95559	0.92801
Learning Rate: 10^−4^, Loss Function: categorical cross-entropy						
Backbone	0.95	0.95	0.95606	0.99115	0.94243	0.91449
Proposed Method	0.97	0.97	0.96053	0.99558	0.96898	0.96855
Learning Rate: 10^−3^, Loss Function: categorical cross-entropy						
Backbone	0.78	0.78	0.67975	0.89333	0.8266	0.72973
Proposed Method	0.94	0.94	0.90158	0.97534	0.94884	0.94354

**Table 9:** Classification results of the backbone vs. proposed model based on PR curve using Data_A.

Precision-Recall (AP)	Micro-Average	COVID-19	Lung Opacity	Normal	Pneumonia
Learning Rate: 10^−4^, Loss Function: categorical_smooth_loss					
Backbone	0.85	0.92	0.84	0.77	0.87
Proposed Method	0.87	0.95	0.93	0.77	0.84
Learning Rate: 10^−3^, Loss Function: categorical_smooth_loss					
Backbone	0.80	0.83	0.81	0.75	0.86
Proposed Method	0.89	0.92	0.95	0.83	0.87
Learning Rate: 10^−4^, Loss Function: categorical cross-entropy					
Backbone	0.88	0.92	0.95	0.81	0.85
Proposed Method	0.93	0.94	0.97	0.89	0. 93
Learning Rate: 10^−3^, Loss Function: categorical cross-entropy					
Backbone	0.54	0.50	0.64	0.52	0.59
Proposed Method	0.90	0.97	0.95	0.80	0.87

**Table 10:** Classification results of the backbone vs. proposed model using Data_B.

Models	Accuracy (%)	Sensitivity (%)			Specificity (%)		Precision (%)		F1_score			AUC
Learning Rate: 10^−4^, Loss Function: categorical_smooth_loss												
Proposed Model	0.9819	0.9729			0.9864		0.9729		0.9729			0.9810
Backbone	0.9720	0.9580			0.9790		0.9583		0.9580			0.9686
ROC	Macro-Average Area		Micro-Average Area			Class 0 Area		Class 1 Area			Class 2 Area	
Proposed Model	0.98		0.98			0.9842		0.9700			0.9848	
Backbone	0.97		0.97			0.9771		0.9541			0.9741	
Average Precision	Micro-Average Precision-Recall			Class 0 AP			Class 1 AP			Class 2 AP		
Proposed Model	0.96			0.9796			0.9606			0.9785		
Backbone	0.93			0.9461			0.9077			0.9574		

**Table 11:** Proposed model classification performance comparison with state-of-the-art models using Data_A.

Reference	Year	Model	Accuracy	Precision	Sensitivity	F1_score
Khan et al. (Strategy 1) [73]	2022	EfficientNetB1	92	91.75	94.50	92.75
		NasNetMobile	89.30	89.25	91.75	91
		MobileNetV2	90.03	92.25	92	91.75
Khan et al. (Strategy 2) [73]	2022	EfficientNetB1	96.13	97.25	96.50	97.50
		NasNetMobile	94.81	95.50	95	95.25
		MobileNetV2	93.96	94.50	95	94.50
Mondal et al. [71]	2022	Local Global Attention Network	95.87	95.56	95.99	95.74
Shi et al. [72]	2021	Teacher Student Attention	91.38	91.65	90.86	91.24
Li et al. [70]	2021	Mag-SD	92.35	92.50	92.20	92.34
Khan et al. [69]	2020	CoroNet	89.6	90.0	96.4	89.8
Shi et al. [72]	2020	COVIDNet	90.78	91.1	90.56	90.81
**Ours**	**2022**		**98.00**	**96.21**	**96.02**	**96.03**

**Table 12:** Proposed model classification performance comparison with state-of-the-art models using Data_B.

Reference	Year	Architecture	Accuracy	Precision	Recall	F1_score
Naralasetti et al. [74]	2021	Deep CNN	91%	-	-	-
Dokur et al. [75]	2020	CNN Ensemble	78% 75%	80% 77%	78% 75%	78% 75%
Hammoudi et al. [36]	2020	VGG19 ResNet+RNN1 ResNet+RNN2 DenseNet169	83% 78% 80% 96%	- - - -	- - - -	- - - -
Windodo et al. [76]	2021	UBNetV1 UBNetV2	88% 88%	89% 89%	86% 85%	86% 86%
Kermany et al. [77]	2021	AutoML	86%	82%	84%	84%
Ibrahim et al. [78]	2020	AlexNet	97.40%	-	-	-
**Ours**	**2022**		**98.19%**	**97.29%**	**97.29%**	**97.29%**

## Data Availability

The dataset used in this paper is public and can be accessed via https://www.kaggle.com/datasets/tawsifurrahman/covid19-radiography-database (accessed on 12 July 2022) and https://github.com/abeerbadawi/COVID-ChestXray15k-Dataset-Transfer-learning (accessed on 17 July 2022). The TensorFlow/Keras code we used in our experiment is not yet publicly available and will be made so after the publication of the work.

## References

[1] Fong S.J., Dey N., Chaki J. (2021). An Introduction to COVID-19. SpringerBriefs in Applied Sciences and Technology.

[2] Bakare O.O., Gokul A., Keyster M. (2022). Analytical Studies of Antimicrobial Peptides as Diagnostic Biomarkers for the Detection of Bacterial and Viral Pneumonia. Bioengineering.

[3] Padda I., Khehra N., Jaferi U., Parmar M.S. (2020). The Neurological Complexities and Prognosis of COVID-19. SN Compr. Clin. Med..

[4] Chen X., Laurent S., Onur O.A., Kleineberg N.N., Fink G.R., Schweitzer F., Warnke C. (2021). A systematic review of neurological symptoms and complications of COVID-19. J. Neurol..

[5] Bentivegna E., Luciani M., Spuntarelli V., Speranza M.L., Guerritore L., Sentimentale A., Martelletti P. (2020). Extremely Severe Case of COVID-19 Pneumonia Recovered Despite Bad Prognostic Indicators: A Didactic Report. SN Compr. Clin. Med..

[6] Ukwuoma C.C., Zhiguang Q., Heyat M.B.B., Ali L., Almaspoor Z., Monday H.N. (2022). Recent Advancements in Fruit Detection and Classification Using Deep Learning Techniques. Math. Probl. Eng..

[7] Mehrrotraa R., Ansari M.A., Agrawal R., Tripathi P., Heyat M.B.B., Al-Sarem M., Muaad A.Y.M., Nagmeldin W.A.E., Abdelmaboud A., Saeed F. (2022). Ensembling of Efficient Deep Convolutional Networks and Machine Learning Algorithms for Resource Effective Detection of Tuberculosis Using Thoracic (Chest) Radiography. IEEE Access.

[8] Nawabi A.K., Jinfang S., Abbasi R., Iqbal M.S., Heyat M.B.B., Akhtar F., Wu K., Twumasi B.A. (2022). Segmentation of Drug-Treated Cell Image and Mitochondrial-Oxidative Stress Using Deep Convolutional Neural Network. Oxid. Med. Cell. Longev..

[9] Ukwuoma C.C., Heyat M.B.B., Masadeh M., Akhtar F., Zhiguang Q., Bondzie-Selby E., Alshorman O., Alkahtani F. (2021). Image Inpainting and Classification Agent Training Based on Reinforcement Learning and Generative Models with Attention Mechanism. Proceedings of the International Conference on Microelectronics, ICM.

[10] Ukwuoma C.C., Qin Z., Heyat M.B.B., Akhtar F., Bamisile O., Muaad A.Y., Addo D., Al-antari M.A. (2022). A hybrid explainable ensemble transformer encoder for pneumonia identification from chest X-ray images. J. Adv. Res..

[11] Khatri A., Jain R., Vashista H., Mittal N., Ranjan P., Janardhanan R. (2020). Pneumonia identification in chest X-ray images using EMD. Trends Commun. Cloud Big Data.

[12] Angeline R., Mrithika M., Raman A., Warrier P. (2020). Pneumonia Detection and Classification Using Chest X-Ray Images with Convolutional Neural Network. New Trends in Computational Vision and Bio-Inspired Computing—Selected Works Presented at the ICCVBIC 2018.

[13] Rousan L.A., Elobeid E., Karrar M., Khader Y. (2020). Chest X-ray findings and temporal lung changes in patients with COVID-19 pneumonia. BMC Pulm. Med..

[14] Asuntha A., Srinivasan A. (2020). Deep learning for lung Cancer detection and classification. Multimed. Tools Appl..

[15] Ricciardi C., Ponsiglione A.M., Scala A., Borrelli A., Misasi M., Romano G., Russo G., Triassi M., Improta G. (2022). Machine Learning and Regression Analysis to Model the Length of Hospital Stay in Patients with Femur Fracture. Bioengineering.

[16] Sultana A., Begum W., Saeedi R., Rahman K., Bin Heyat M.B., Akhtar F., Son N.T., Ullah H. (2022). Experimental and Computational Approaches for the Classification and Correlation of Temperament (Mizaj) and Uterine Dystemperament (Su’-I-Mizaj Al-Rahim) in Abnormal Vaginal Discharge (Sayalan Al-Rahim) Based on Clinical Analysis Using Support Vector Machine. Complexity.

[17] Teelhawod B.N., Akhtar F., Heyat M.B.B., Tripathi P., Mehrotra R., Asfaw A.B., Shorman O.A., Masadeh M. Machine Learning in E-health: A Comprehensive Survey of Anxiety. Proceedings of the 2021 International Conference on Data Analytics for Business and Industry, ICDABI.

[18] Akhtar F., Heyat M.B.B., Li J.P., Patel P.K., Guragai B. (2020). Role of Machine Learning in Human Stress: A Review. Proceedings of the 2020 17th International Computer Conference on Wavelet Active Media Technology and Information Processing, ICCWAMTIP.

[19] Guragai B., Alshorman O., Masadeh M., Heyat M.B. (2020). Bin A Survey on Deep Learning Classification Algorithms for Motor Imagery. Proceedings of the International Conference on Microelectronics, ICM.

[20] Heyat M.B.B., Lai D., Khan F.I., Zhang Y. (2019). Sleep Bruxism Detection Using Decision Tree Method by the Combination of C4-P4 and C4-A1 Channels of Scalp EEG. IEEE Access.

[21] Bin Heyat M.B., Akhtar F., Abbas S.J., Al-Sarem M., Alqarafi A., Stalin A., Abbasi R., Muaad A.Y., Lai D., Wu K. (2022). Wearable Flexible Electronics Based Cardiac Electrode for Researcher Mental Stress Detection System Using Machine Learning Models on Single Lead Electrocardiogram Signal. Biosensors.

[22] Sultana A., Rahman K., Heyat M.B.B., Sumbul, Akhtar F., Muaad A.Y. (2022). Role of Inflammation, Oxidative Stress, and Mitochondrial Changes in Premenstrual Psychosomatic Behavioral Symptoms with Anti-Inflammatory, Antioxidant Herbs, and Nutritional Supplements. Oxid. Med. Cell. Longev..

[23] Heyat M.B.B., Akhtar F., Khan M.H., Ullah N., Gul I., Khan H., Lai D. (2020). Detection, Treatment Planning, and Genetic Predisposition of Bruxism: A Systematic Mapping Process and Network Visualization Technique. CNS Neurol. Disord. Drug Targets.

[24] Heyat M.B.B., Akhtar F., Khan A., Noor A., Benjdira B., Qamar Y., Abbas S.J., Lai D. (2020). A novel hybrid machine learning classification for the detection of bruxism patients using physiological signals. Appl. Sci..

[25] Heyat M.B.B., Akhtar F., Ansari M.A., Khan A., Alkahtani F., Khan H., Lai D. (2020). Progress in Detection of Insomnia Sleep Disorder: A Comprehensive Review. Curr. Drug Targets.

[26] Vaswani A., Shazeer N., Parmar N., Uszkoreit J., Jones L., Gomez A.N., Kaiser Ł., Polosukhin I. Attention is all you need. Proceedings of the Advances in Neural Information Processing Systems.

[27] Ott M., Edunov S., Grangier D., Auli M. (2018). Scaling Neural Machine Translation. Proceedings of the WMT 2018—3rd Conference on Machine Translation.

[28] Fedus W., Zoph B., Shazeer N. (2022). Switch Transformers: Scaling to Trillion Parameter Models with Simple and Efficient Sparsity. J. Mach. Learn. Res..

[29] Chaudhari S., Mithal V., Polatkan G., Ramanath R. (2021). An Attentive Survey of Attention Models. ACM Trans. Intell. Syst. Technol..

[30] Albahli S., Rauf H.T., Arif M., Nafis M.T., Algosaibi A. (2020). Identification of thoracic diseases by exploiting deep neural networks. Comput. Mater. Contin..

[31] Elshennawy N.M., Ibrahim D.M. (2020). Deep-Pneumonia Framework Using Deep Learning Models Based on Chest X-ray Images. Diagnostics.

[32] Wang X., Peng Y., Lu L., Lu Z., Bagheri M., Summers R.M. ChestX-ray8: Hospital-scale chest X-ray database and benchmarks on weakly-supervised classification and localization of common thorax diseases. Proceedings of the 2017 IEEE Conference on Computer Vision and Pattern Recognition (CVPR).

[33] Talo M. (2019). Pneumonia detection from radiography images using convolutional neural networks. Proceedings of the 27th Signal Processing and Communications Applications Conference, SIU.

[34] Varshni D., Thakral K., Agarwal L., Nijhawan R., Mittal A. (2019). Pneumonia Detection Using CNN based Feature Extraction. Proceedings of the 2019 3rd IEEE International Conference on Electrical, Computer and Communication Technologies, ICECCT.

[35] Stephen O., Sain M., Maduh U.J., Jeong D.U. (2019). An Efficient Deep Learning Approach to Pneumonia Classification in Healthcare. J. Healthc. Eng..

[36] Hammoudi K., Benhabiles H., Melkemi M., Dornaika F., Arganda-Carreras I., Collard D., Scherpereel A. (2021). Deep Learning on Chest X-ray Images to Detect and Evaluate Pneumonia Cases at the Era of COVID-19. J. Med. Syst..

[37] Sirazitdinov I., Kholiavchenko M., Mustafaev T., Yixuan Y., Kuleev R., Ibragimov B. (2019). Deep neural network ensemble for pneumonia localization from a large-scale chest x-ray database. Comput. Electr. Eng..

[38] Liang G., Zheng L. (2020). A transfer learning method with deep residual network for pediatric pneumonia diagnosis. Comput. Methods Programs Biomed..

[39] Chouhan V., Singh S.K., Khamparia A., Gupta D., Tiwari P., Moreira C., Damaševičius R., de Albuquerque V.H.C. (2020). A novel transfer learning based approach for pneumonia detection in chest X-ray images. Appl. Sci..

[40] Siddiqi R. (2019). Automated pneumonia diagnosis using a customized sequential convolutional neural network. ACM International Conference Proceeding Series, Proceedings of the 2019 3rd International Conference on Deep Learning Technologies, Xiamen, China, 5–7 July 2019.

[41] Jain R., Nagrath P., Kataria G., Kaushik V.S., Hemanth D.J. (2020). Pneumonia detection in chest X-ray images using convolutional neural networks and transfer learning. Meas. J. Int. Meas. Confed..

[42] Nayak S.R., Nayak D.R., Sinha U., Arora V., Pachori R.B. (2021). Application of deep learning techniques for detection of COVID-19 cases using chest X-ray images: A comprehensive study. Biomed. Signal. Process. Control..

[43] Ozturk T., Talo M., Yildirim E.A., Baloglu U.B., Yildirim O., Acharya U.R. (2020). Automated detection of COVID-19 cases using deep neural networks with X-ray images. Comput. Biol. Med..

[44] Das A.K., Kalam S., Kumar C., Sinha D. (2021). TLCoV—An automated COVID-19 screening model using Transfer Learning from chest X-ray images. Chaos Solitons Fractals.

[45] Monshi M.M.A., Poon J., Chung V., Monshi F.M. (2021). CovidXrayNet: Optimizing data augmentation and CNN hyperparameters for improved COVID-19 detection from CXR. Comput. Biol. Med..

[46] Rajpal S., Lakhyani N., Singh A.K., Kohli R., Kumar N. (2021). Using handpicked features in conjunction with ResNet-50 for improved detection of COVID-19 from chest X-ray images. Chaos Solitons Fractals.

[47] Kumar A., Tripathi A.R., Satapathy S.C., Zhang Y.D. (2022). SARS-Net: COVID-19 detection from chest x-rays by combining graph convolutional network and convolutional neural network. Pattern Recognit..

[48] Narin A., Kaya C., Pamuk Z. (2021). Automatic detection of coronavirus disease (COVID-19) using X-ray images and deep convolutional neural networks. Pattern Anal. Appl..

[49] Wang L., Lin Z.Q., Wong A. (2020). COVID-Net: A tailored deep convolutional neural network design for detection of COVID-19 cases from chest X-ray images. Sci. Rep..

[50] Sohail A., Yu Z., Nutini A. (2022). COVID-19 Variants and Transfer Learning for the Emerging Stringency Indices. Neural Process. Lett..

[51] Wang Y., Huang R., Song S., Huang Z., Huang G. (2021). Not All Images are Worth 16x16 Words: Dynamic Transformers for Efficient Image Recognition. Adv. Neural Inf. Process. Syst..

[52] Gao Z., Xie J., Wang Q., Li P. Global Second-Order Pooling Convolutional Networks. Proceedings of the 2019 IEEE/CVF Conference on Computer Vision and Pattern Recognition (CVPR).

[53] Chowdhury M.E.H., Rahman T., Khandakar A., Mazhar R., Kadir M.A., Mahbub Z.B., Islam K.R., Khan M.S., Iqbal A., Emadi N.A. (2020). Can AI Help in Screening Viral and COVID-19 Pneumonia?. IEEE Access.

[54] Badawi A., Elgazzar K. (2021). Detecting Coronavirus from Chest X-rays Using Transfer Learning. COVID.

[55] Ioffe S., Szegedy C. Batch normalization: Accelerating deep network training by reducing internal covariate shift. Proceedings of the 32nd International Conference on Machine Learning.

[56] He K., Zhang X., Ren S., Sun J. Deep residual learning for image recognition. Proceedings of the IEEE Computer Society Conference on Computer Vision and Pattern Recognition.

[57] Ullah H., Heyat M.B.B., Akhtar F., Sumbul, Muaad A.Y., Islam M.S., Abbas Z., Pan T., Gao M., Lin Y. (2022). An End-to-End Cardiac Arrhythmia Recognition Method with an Effective DenseNet Model on Imbalanced Datasets Using ECG Signal. Comput. Intell. Neurosci..

[58] Neurohive (2018). VGG16—Convolutional Network for Classification and Detection. https://neurohive.io/en/popular-networks/vgg16/.

[59] Szegedy C., Liu W., Jia Y., Sermanet P., Reed S., Anguelov D., Erhan D., Vanhoucke V., Rabinovich A. Going Deeper with Convolutions. Proceedings of the IEEE Conference on Computer Vision and Pattern Recognition.

[60] Ali L., He Z., Cao W., Rauf H.T., Imrana Y., Bin Heyat M.B. (2021). MMDD-Ensemble: A Multimodal Data–Driven Ensemble Approach for Parkinson’s Disease Detection. Front. Neurosci..

[61] Tripathi P., Ansari M.A., Gandhi T.K., Mehrotra R., Heyat M.B.B., Akhtar F., Ukwuoma C.C., Muaad A.Y., Kadah Y.M., Al-Antari M.A. (2022). Ensemble Computational Intelligent for Insomnia Sleep Stage Detection via the Sleep ECG Signal. IEEE Access.

[62] Ullah H., Bin Heyat M.B., Alsalman H., Khan H.M., Akhtar F., Gumaei A., Mehdi A., Muaad A.Y., Islam M.S., Ali A. (2022). An Effective and Lightweight Deep Electrocardiography Arrhythmia Recognition Model Using Novel Special and Native Structural Regularization Techniques on Cardiac Signal. J. Healthc. Eng..

[63] Iqbal M.S., Abbasi R., Heyat M.B.B., Akhtar F., Abdelgeliel A.S., Albogami S., Fayad E., Iqbal M.A. (2022). Recognition of mRNA N4 Acetylcytidine (ac4C) by Using Non-Deep vs. Deep Learning. Appl. Sci..

[64] AlShorman O., Masadeh M., Heyat M.B.B., Akhtar F., Almahasneh H., Ashraf G.M., Alexiou A. (2022). Frontal lobe real-time EEG analysis using machine learning techniques for mental stress detection. J. Integr. Neurosci..

[65] Tripathi P., Ansari M.A., Akhtar F., Bin Heyat M.B., Mehrotra R., Yatoo A.H., Teelhawod B.N., Asfaw A.B., Baig A.A. (2022). Automatic Epileptic Seizure Detection Based on the Discrete Wavelet Transform Approach Using an Artificial Neural Network Classifier on the Scalp Electroencephalogram Signal. Comput. Intell. Healthc. Appl..

[66] Ukwuoma C.C., Urama G.C., Qin Z., Bin Heyat M.B., Mohammed Khan H., Akhtar F., Masadeh M.S., Ibegbulam C.S., Delali F.L., AlShorman O. Boosting Breast Cancer Classification from Microscopic Images Using Attention Mechanism. Proceedings of the 2022 International Conference on Decision Aid Sciences and Applications (DASA).

[67] Ukwuoma C.C., Zhiguang Q., Bin Heyat M.B., Mohammed Khan H., Akhtar F., Masadeh M.S., Bamisile O., AlShorman O., Nneji G.U. Detection of Oral Cavity Squamous Cell Carcinoma from Normal Epithelium of the Oral Cavity Using Microscopic Images. Proceedings of the 2022 International Conference on Decision Aid Sciences and Applications (DASA).

[68] Chola C., Muaad A.Y., Bin Heyat M.B., Benifa J.V.B., Naji W.R., Hemachandran K., Mahmoud N.F., Samee N.A., Al-Antari M.A., Kadah Y.M. (2022). BCNet: A Deep Learning Computer-Aided Diagnosis Framework for Human Peripheral Blood Cell Identification. Diagnostics.

[69] Khan A.I., Shah J.L., Bhat M.M. (2020). CoroNet: A deep neural network for detection and diagnosis of COVID-19 from chest x-ray images. Comput. Methods Programs Biomed..

[70] Li J., Wang Y., Wang S., Wang J., Liu J., Jin Q., Sun L. (2021). Multiscale Attention Guided Network for COVID-19 Diagnosis Using Chest X-Ray Images. IEEE J. Biomed. Health Inform..

[71] Mondal A.K. (2022). COVID-19 prognosis using limited chest X-ray images. Appl. Soft Comput..

[72] Shi W., Tong L., Zhu Y., Wang M.D. (2021). COVID-19 Automatic Diagnosis with Radiographic Imaging: Explainable Attention Transfer Deep Neural Networks. IEEE J. Biomed. Health Inform..

[73] Khan E., Rehman M.Z.U., Ahmed F., Alfouzan F.A., Alzahrani N.M., Ahmad J. (2022). Chest X-ray Classification for the Detection of COVID-19 Using Deep Learning Techniques. Sensors.

[74] Naralasetti V., Shaik R.K., Katepalli G., Bodapati J.D. (2021). Deep learning models for Pneumonia identification and classification based on X-ray images. Trait. du Signal..

[75] Darici M.B., Dokur Z., Olmez T. (2020). Pneumonia detection and classification using deep learning on chest x-ray images. Int. J. Intell. Syst. Appl. Eng..

[76] Widodo C.S., Naba A., Mahasin M.M., Yueniwati Y., Putranto T.A., Patra P.I. (2022). UBNet: Deep learning-based approach for automatic X-ray image detection of pneumonia and COVID-19 patients. J. Xray. Sci. Technol..

[77] Kermany D.S., Goldbaum M., Cai W., Valentim C.C.S., Liang H., Baxter S.L., McKeown A., Yang G., Wu X., Yan F. (2018). Identifying Medical Diagnoses and Treatable Diseases by Image-Based Deep Learning. Cell.

[78] Ibrahim A.U., Ozsoz M., Serte S., Al-Turjman F., Yakoi P.S. (2021). Pneumonia Classification Using Deep Learning from Chest X-ray Images During COVID-19. Cognit. Comput..

[79] Panwar H., Gupta P.K., Siddiqui M.K., Morales-Menendez R., Singh V. (2020). Application of deep learning for fast detection of COVID-19 in X-Rays using nCOVnet. Chaos Solitons Fractals.

